# P-1011. Real-World Antifungal Therapy Patterns in US Patients with Invasive Aspergillosis: It’s Complicated

**DOI:** 10.1093/ofid/ofae631.1201

**Published:** 2025-01-29

**Authors:** Barbara D Alexander, Melissa D Johnson, Mark Bresnik, Ruthwik Anupindi, Lia Pizzicato, Mitchell DeKoven, Belinda Lovelace, Craig I Coleman

**Affiliations:** Duke University School of Medicine, Durham, North Carolina; Duke University, Durham, North Carolina; F2G, Ltd., Princeton, New Jersey; IQVIA, Falls Church, Virginia; IQVIA, Falls Church, Virginia; IQVIA, Falls Church, Virginia; F2G, Inc., Princeton, New Jersey; University of Connecticut, Storrs, Connecticut

## Abstract

**Background:**

The complexity of antifungal therapy (AFT) in Invasive Aspergillosis (IA) can complicate care. We sought to better understand AFT changes that occur in IA across both in- and outpatient settings of care.

**Table 1.**
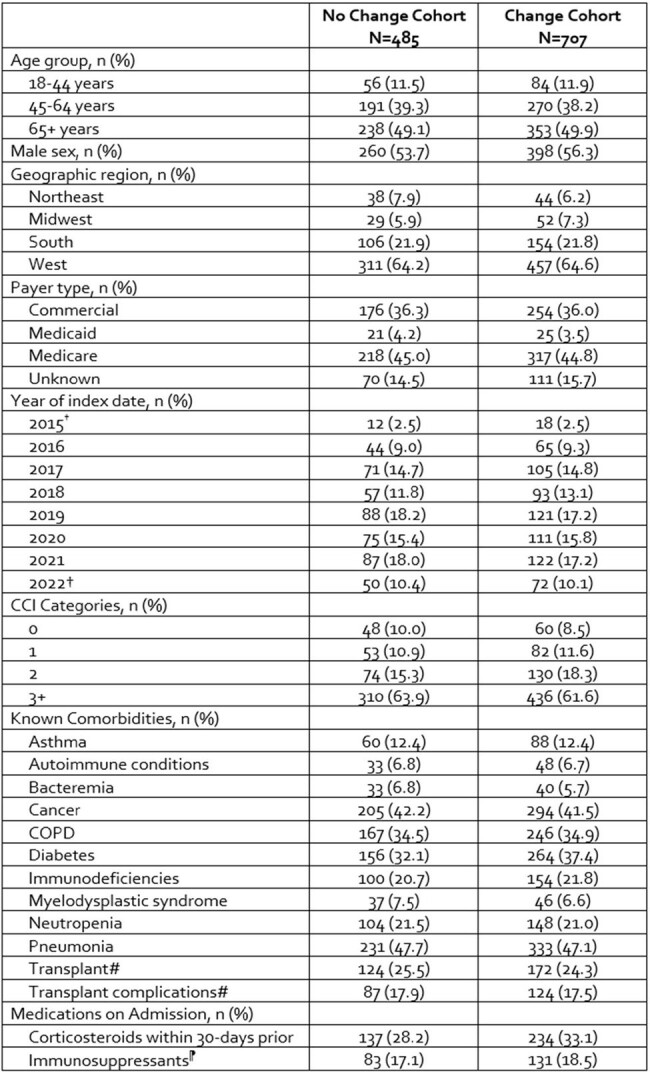
Baseline Characteristics of Patients Who Changed or Did Not Change Their Invasive Aspergillosis Antifungal Therapy*

CCI=Charlson comorbidity index; COPD=chronic obstructive pulmonary disease; IA= invasive Aspergillosis

†Partial years data were available in IQVIA New Data Warehouse

#Solid organ or hematopoietic cell transplant

¶Azathioprine, cyclophosphamide, cyclosporine, methotrexate, mycophenolate, tacrolimus, voclosporin

*The following covariates were used to calculate propensity scores for Inverse probability of treatment weighting: age group, geographic region, payer type, year of index, asthma, autoimmune conditions, bacteremia, COPD, pneumonia, immunodeficiencies, myelodysplastic syndrome, transplant history, immunosuppressants, cancer, neutropenia, and total healthcare costs.

∥All covariates were well balanced as indicated by an SMD <0.1 except corticosteroids which was marginally imbalanced with an SMD of 0.108

**Methods:**

This retrospective study was performed using the IQVIA New Data Warehouse (hospital, outpatient, and prescription claims) from Oct 2015-Nov 2022. The index inpatient stay was identified by IA ICD-10 codes. Patients were followed from index hospitalization until end of the study period (Dec 31, 2022), lack of medical or pharmacy activity in the database, or death (whichever occurred first). Patients were stratified by whether they ‘changed’ (defined as discontinuation with restart of a non-first line (L) AFT, switch or modification) or ‘did not change’ their AFT during the post-index period. Inverse probability of treatment weighting was used to minimize confounding due to baseline differences between the change and no change cohorts. Data were reported as frequencies or median with interquartile range.

**Figure 1.**
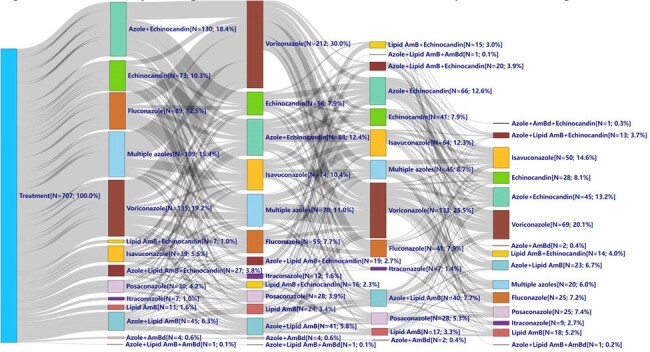
Sankey Diagram of IA Patients Who Required Changes in Antifungal Therapy

**Results:**

Among 1,192 IA adults, 707 (59.3%) experienced changes to their AFT (60% modified existing AFT, 22.1% stopped 1L AFT and initiated AFT for 2L, and 18% directly switched to a different AFT) **(Table 1)**. Among those who changed AFT, mold active azole use predominated, with voriconazole the most used across all AFT lines (37.3-49.3%), followed by isavuconazole (19.3-26.7%) **(Figure 1)**. Echinocandin use varied between 25.3%-33.6% over 1-4L and amphotericin B use steadily increased over the lines of therapy (13.4-20.7%). Among the 485 (40.6%) patients that completed AFT with no changes, most received azole (62.8% voriconazole, 15.2% isavuconazole) monotherapy. Patients who changed AFT had shorter time on treatment for 1-3L (5-8 days) than those who did not change (11-21 days) (p< 0.047).

**Conclusion:**

Based on this snapshot of IA treatment patterns, managing AFT for patients with IA is complicated. Most patients require changes to their AFT. Future studies should examine challenges associated with IA treatment and which factors contribute to AFT changes, such as drug toxicities or interactions and refractory infection. Novel oral antifungals that reduce the need for AFT changes when IA patients have limited options are needed.

**Disclosures:**

**Barbara D. Alexander, MD**, Basilea: Advisor/Consultant|F2G: Advisor/Consultant|F2G: Grant/Research Support|HealthTrackRx: Advisor/Consultant|HealthTrackRx: Board Member|Scynexis: Grant/Research Support|TFF Pharmaceuticals: Advisor/Consultant **Melissa D. Johnson, PharmD MHS AAHIVP**, Biomeme: Licensed Technology|Scynexis, Inc: Grant/Research Support|UpToDate: Author Royalties **Mark Bresnik, MD**, F2G Ltd: Employee **Ruthwik Anupindi, PhD**, F2G Ltd: Grant/Research Support|IQVIA: Employee **Lia Pizzicato, MPH**, F2G Ltd: Grant/Research Support|IQVIA: Employee **Mitchell DeKoven, PhD**, F2G Ltd: Grant/Research Support|IQVIA: Employee **Belinda Lovelace, PharmD, MS, MJ**, F2G, Inc.: Employee **Craig I. Coleman, PharmD**, F2G Ltd: Advisor/Consultant|F2G Ltd: Grant/Research Support

